# Supplementary material for: Endometrial Cancer Detection Using a Cervical DNA Methylation Assay (MPap) in Women with Abnormal Uterine Bleeding: A Multicenter Hospital-Based Validation Study
Source: Cancers (Basel). 2022 Sep 5;14(17):4343. doi: 10.3390/cancers14174343 (PMC9454900; doi:10.3390/cancers14174343)
Supplement: Supplementary file 1 [file cancers-14-04343-s001.zip › cancers-1877307-supplementary.pdf]

SUPPLEMENTAL INFORMATION

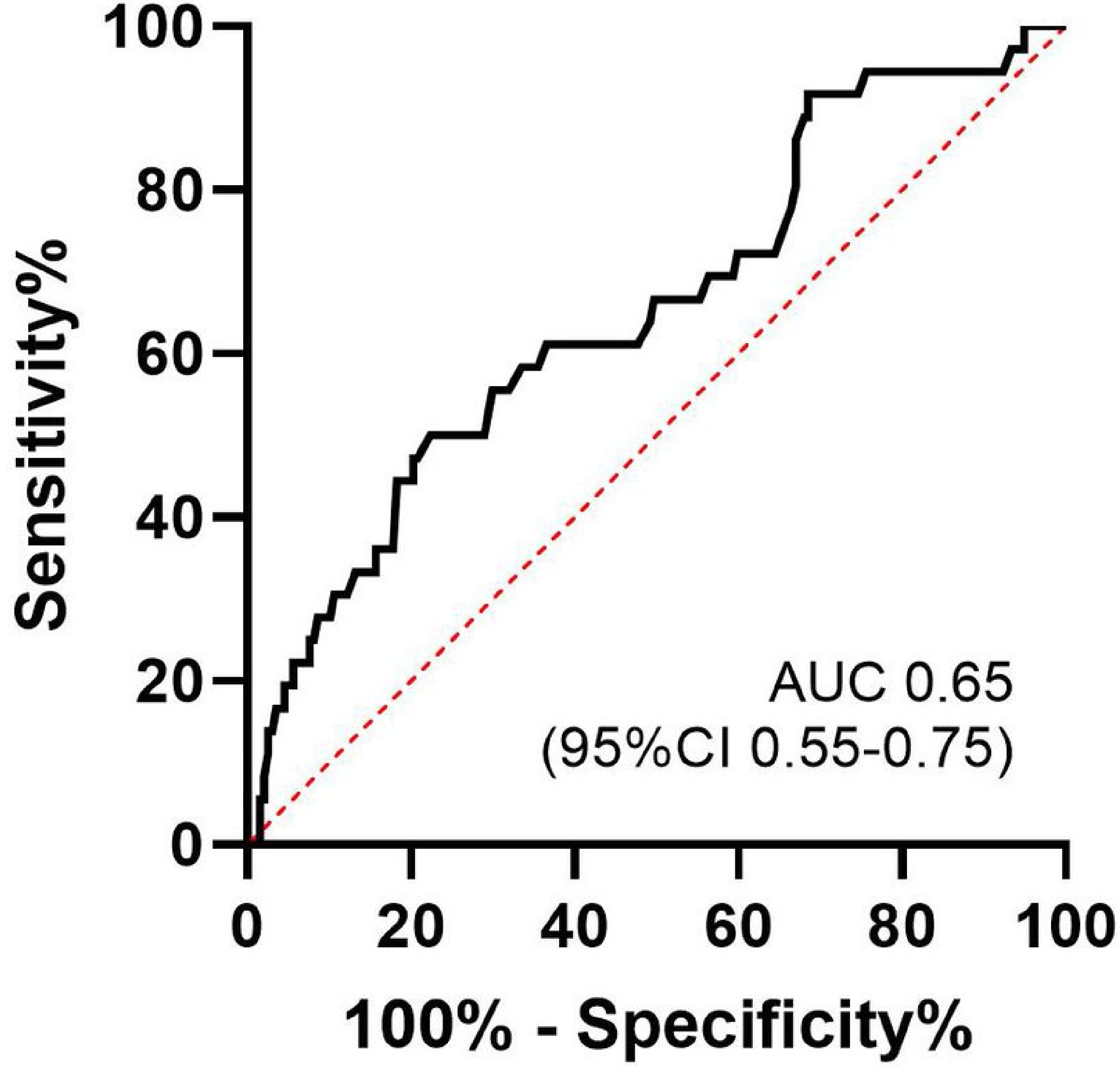

Figure S1. The AUC for different ET thicknesses in detection of EC.

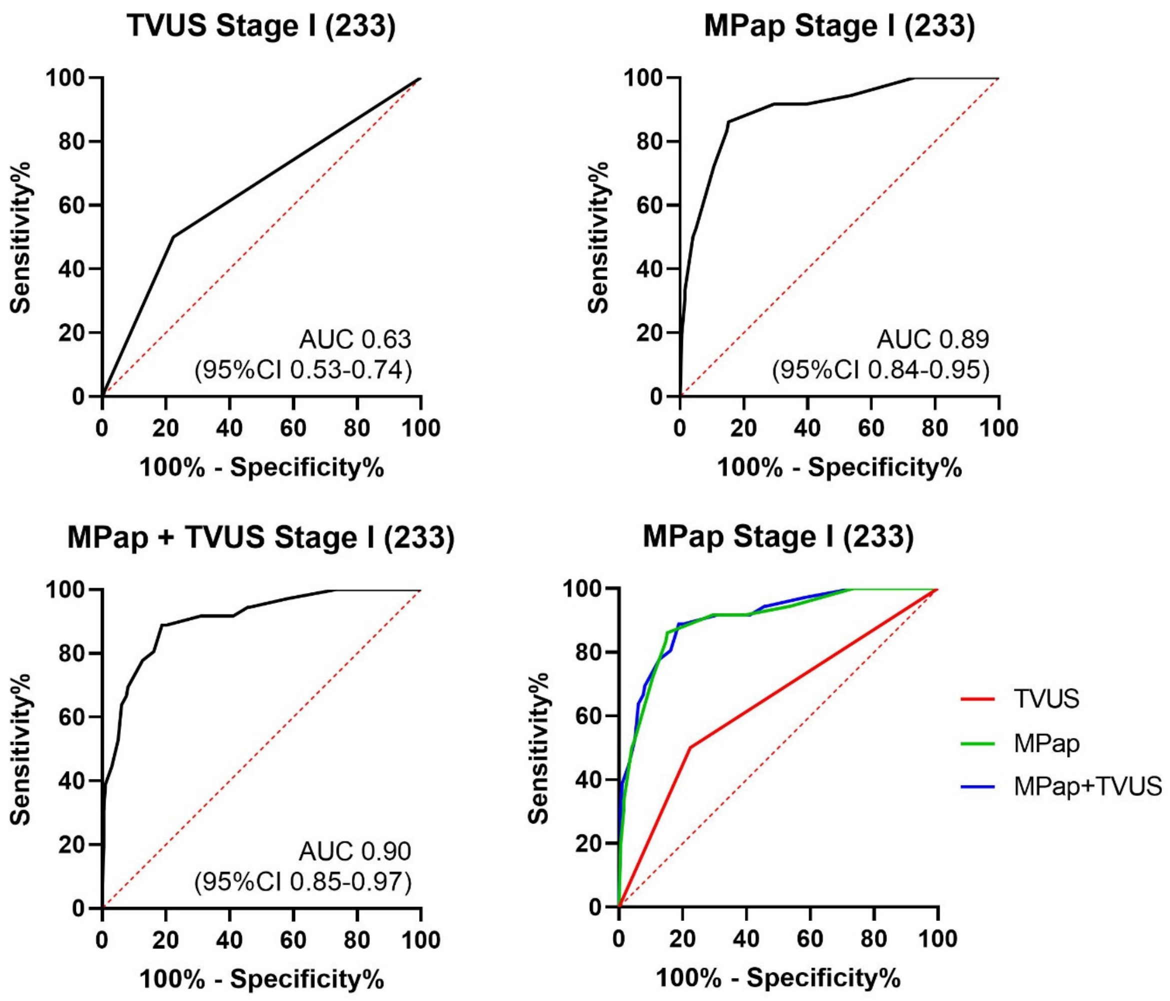

Figure S2. The complement of MPap assay with or without TVUS in stage 1.

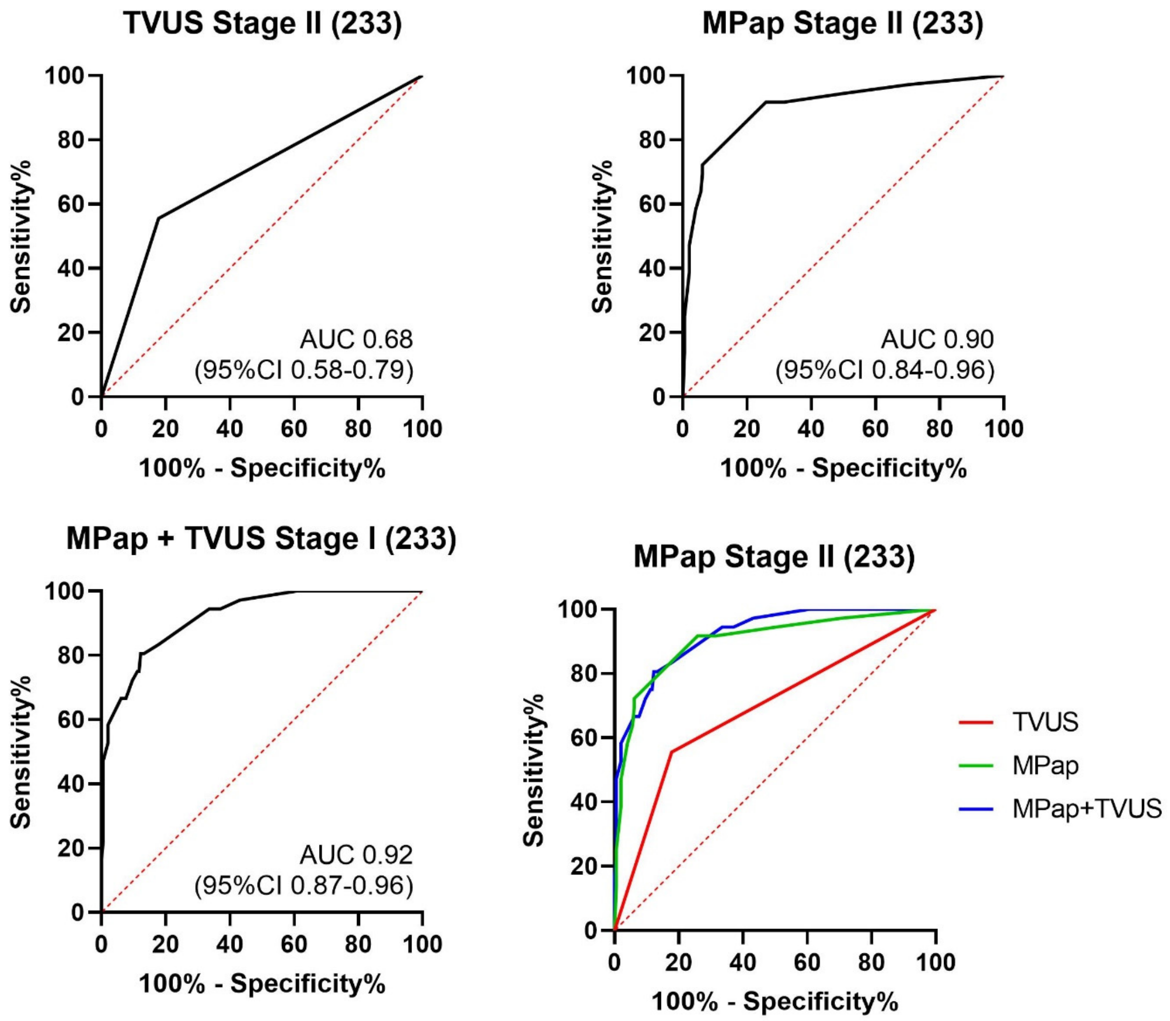

Figure S3. The complement of MPap assay with or without TVUS in stage 2.

Table S1. Classification of endometrial pathology.

Normal physiological change (Normal)	
	Proliferative phase of the endometrium
	Secretory phase of the endometrium
	Menstrual phase of the endometrium
	Atrophic postmenopausal endometrium
Benign endometrial lesions (Benign)	
	Endometrial metaplasia
	Specific or nonspecific endometritis
	Endometrial polyp
	Submucosal leiomyoma
	Disordered proliferative endometrium
Precancerous change (Hyperplasia)	
	Endometrial hyperplasia
	Atypical endometrial hyperplasia
	Endometrial intraepithelial neoplasia
Endometrial cancer (EC)	
	Endometrioid adenocarcinoma
	Carcinosarcoma
	Clear cell adenocarcinoma
	Mixed carcinoma
	Serous adenocarcinoma
	Undifferentiated carcinoma
	Others

Table S2. Characteristics of EC.

Characteristics		*Stage 1**	*Stage 2**	*P value*
		n=42	*n=39*	
Cancer Type	*I*	37 (88)	33 (85)	0.6480
	II	5 (12)	6 (15)	
Cancer Stage	*I*	29 (69)	29 (74)	0.3210
	II	4 (10)	1 (3)	
	III	7 (17)	7 (18)	
	IV	2 (5)	2 (5)	
Cancer Grade	*1*	16 (38)	17 (44)	0.8110
	2	18 (43)	14 (36)	
	3	8 (19)	8 (21)	

* Data are shown as n (%) unless otherwise indicated.

Table S3. MPap values of the participants.

Cohort		*Normal*	*Benign*	*Precancerous*	*EC*	*P Value*
Stage 1	*n*	138	45	24	42	
	*MPap value**	-3.3(1.6)	-3.1(1.7)	-2.9(1.8)	0.2(1.8)	<0.0001
Stage 2	*n*	86	83	37	39	
	*MPap value**	-3.4(1.4)	-3.5(1.5)	-3.7(1.5)	-0.5(1.9)	<0.0001

* Data are shown as Mean (Standard Deviation).

Table S4. Comparison of different thresholds of the MPap value for EC detection.

MPap value	*Stage 1**	*Stage 2**
AUC	0.91 (0.87-0.94)	0.90 (0.84-0.95)
Sen	92.9 (80.5-98.5)	92.5 (82.9-100.0)
Spe	71.5 (64.8-77.5)	73.8 (67.6-79.4)
PPV	39.8 (34.4-45.5)	40.2 (30.8-50.5)
NPV	98.0 (94.3-99.3)	98.1 (95.8-100.0)

* Data % are within the 95% CIs unless otherwise indicated.

The cutoff threshold of the MPap value is -2.10.

Sen: sensitivity; Spe: specificity; PPV: positive predictive value; NPV: negative predictive value.

Table S5. MPap performance in centers from northern, central, and southern Taiwan.

Centers	*Subtotal (n)*	*EC (n)*	*Non-EC (n)*	*Sen (%)*	*Spe (%)*	*P Value*
Northern	236	33	203	90.9	73.4	0.39
Central	103	6	97	83.3	70.1	
Southern	152	42	110	95.2	72.7	

EC, endometrial cancer; Sen: sensitivity; Spe: specificity.

Table S6. Performance of the MPap test for the detection of different histological types of EC.

Histological type	*Stage 1 MPap high risk**	*Stage 2 MPap high risk**
Endometrioid adenocarcinoma	35/37 (95%)	30/33 (91%)
Carcinosarcoma	1/2 (50%)	3/3 (100%)
Clear cell adenocarcinoma	1/1 (100%)	0
Desmoplastic small round cell tumor	0	1/1 (100%)
Mixed carcinoma	2/2 (100%)	0
Serous adenocarcinoma	0	1/1 (100%)
Undifferentiated carcinoma	0	1/1 (100%)

*Data are shown as n or n (%) of the EC cases.

Table S7. The frequency of diagnosing EC among IVPs from the Taiwan Cancer Registry Database.

Age		*NHI data*	*MPap value*	
		n	*High risk*	*Low risk*
50 years+	*IVPs*	17752	5393	12359
	EC	1119	1035	84
	NonEC	16633	4358	12275
	IVPs/per EC	15.9	5.2	
	Triage IVPs (%)			69.1
40-50 years	*IVPs*	20951	5650	15301
	EC	242	224	18
	NonEC	20709	5426	15283
	IVPs/per EC	86.6	25.2	
	Triage IVPs (%)			72.9

NHI: Taiwan's National Health Insurance; IVPs: invasive procedures; EC: endometrial cancer.
